# Incidental Finding of a Popliteal Artery Aneurysm Identified by Duplex Ultrasonography During Deep Vein Thrombosis Screening

**DOI:** 10.7759/cureus.94030

**Published:** 2025-10-07

**Authors:** Shinichi Tanaka, Takahiro Ohmine, Takashi Maeda

**Affiliations:** 1 Department of Surgery, Hiroshima Red Cross Hospital and Atomic-Bomb Survivors Hospital, Hiroshima, JPN

**Keywords:** abdominal aortic aneurysm, duplex ultrasonography, endovascular aortic repair (evar), open surgery, popliteal artery aneurysm

## Abstract

Here, we describe a 78-year-old man in whom a right popliteal artery aneurysm (PAA) was detected incidentally during duplex ultrasonography screening for deep venous thrombus before surgery for prostate cancer. He underwent PAA repair while asymptomatic. In our institution, 2,264 patients underwent screening for deep venous thrombosis due to abnormal circulating D-dimer concentrations before surgery under general anesthesia between 2019 and 2023. Because PAA is a rare disease and its diagnosis before the onset of limb-threatening complications is beneficial for patients, it is necessary to share such findings with colleagues even when screening for diseases other than PAA.

## Introduction

Popliteal artery aneurysm (PAA) is the most common peripheral arterial aneurysm, representing up to 70% of all peripheral aneurysms [1,2]. The prevalence is estimated to be 0.1-1% in the general population, with a marked male predominance and a peak incidence in the seventh decade of life [1]. Although some PAAs remain asymptomatic, many present with limb-threatening complications, such as acute lower limb ischemia (ALLI) due to thrombosis or distal embolization, with up to 20% of affected patients requiring major amputation if untreated [3,4].

Current guidelines recommend repair for symptomatic PAAs and for asymptomatic aneurysms larger than 20 mm, or when associated with mural thrombus or distal embolization [1]. Treatment strategies include open surgical repair, regarded as the gold standard, and endovascular repair with covered stents, which is increasingly utilized in carefully selected patients [5].

However, the incidental diagnosis of asymptomatic PAAs remains uncommon, as vascular imaging is rarely performed in the absence of symptoms. We report a case of an asymptomatic PAA incidentally detected during routine medical evaluation, underscoring the importance of early recognition and appropriate management of this condition.

## Case presentation

**Figure 1:**
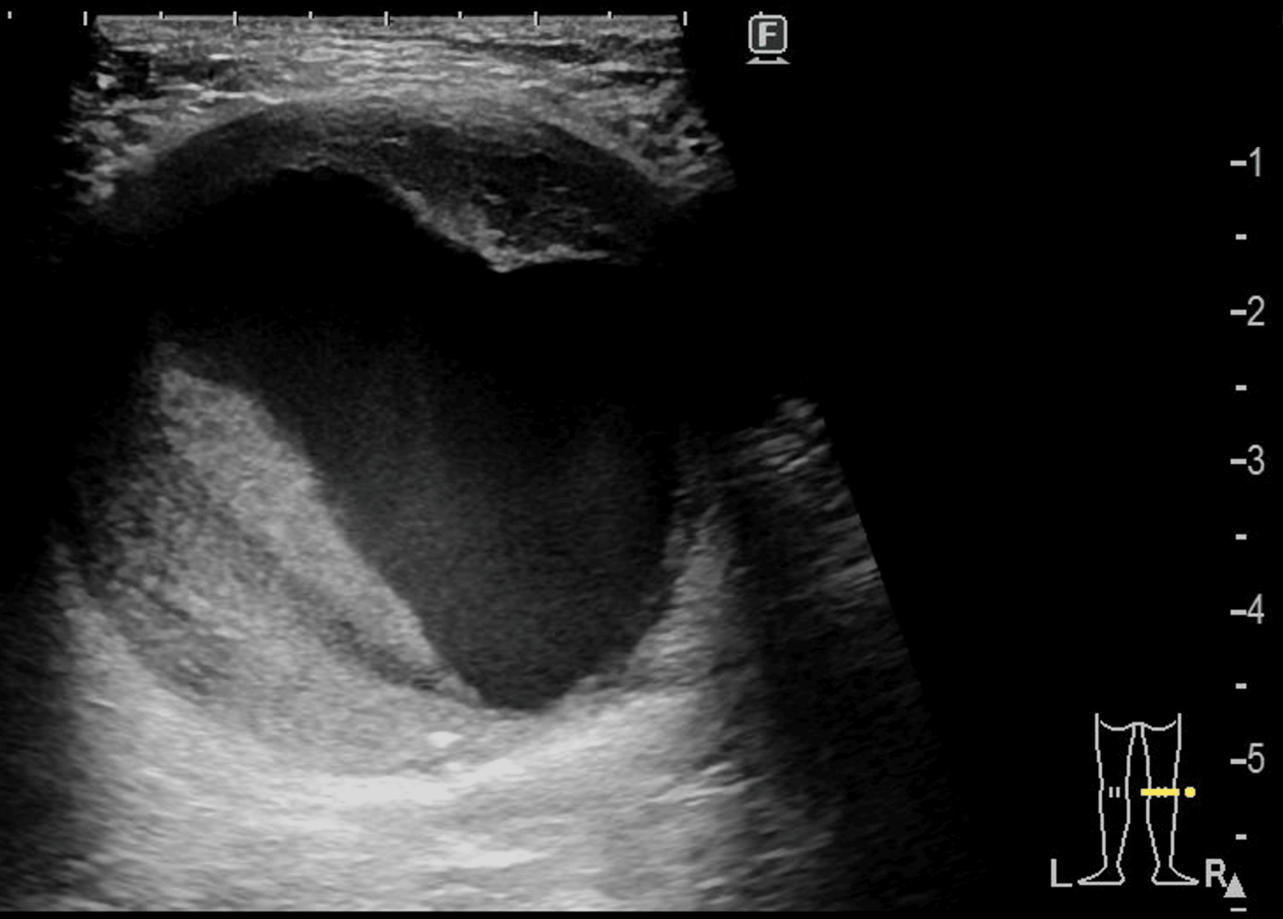
A right popliteal artery aneurysm was an incidental finding on duplex ultrasonography.

**Figure 2:**
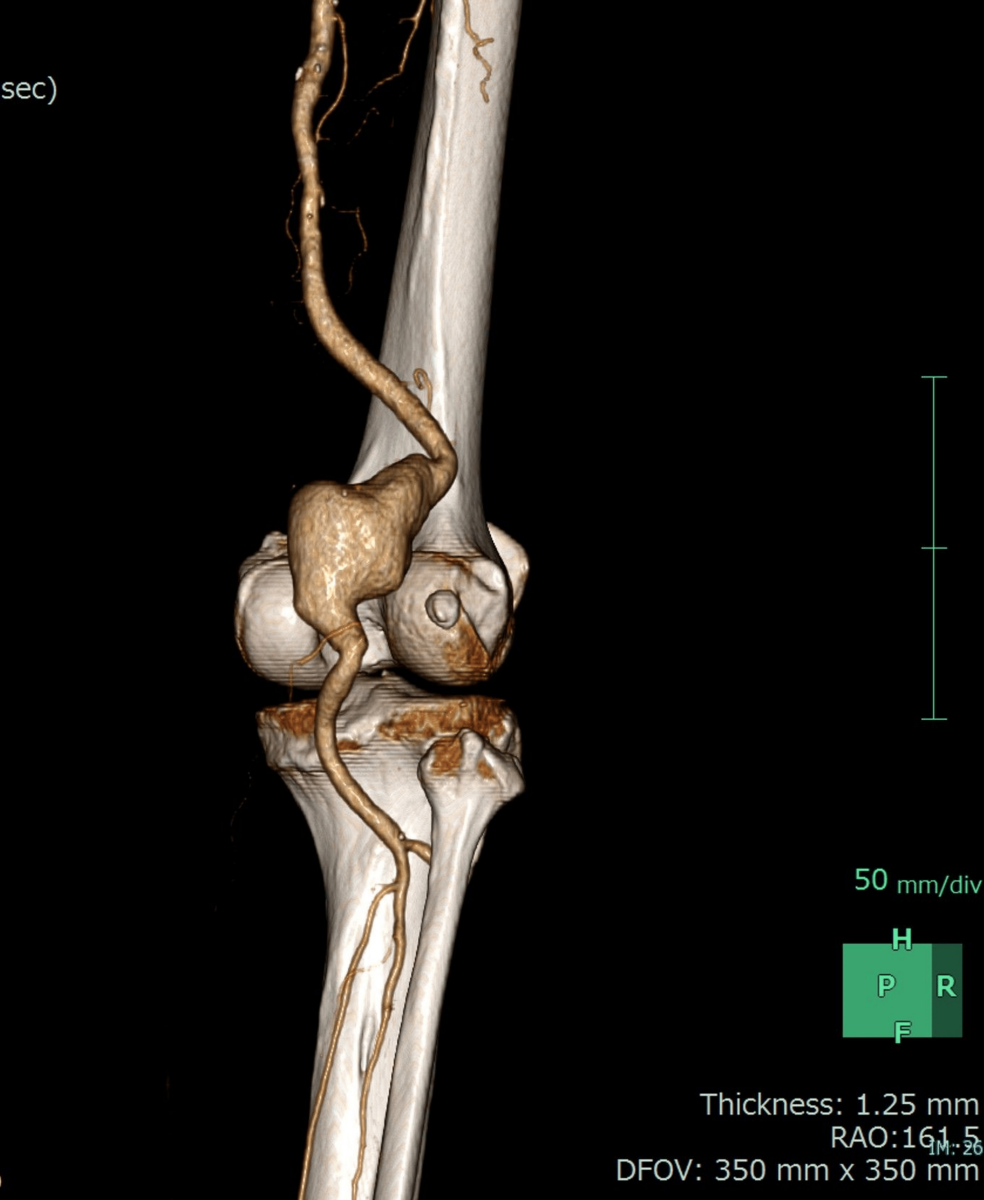
Three-dimensional computed tomography image of the right popliteal artery aneurysm.

A 78-year-old man was diagnosed with prostate cancer in the urology department of the hospital. He was a current smoker and had hypertension. He was scheduled to undergo radical prostatectomy under general anesthesia. Preanesthetic blood screening revealed a D-dimer concentration of 8.5 μg/mL (reference range: <0.5 µg/mL). Therefore, screening for deep venous thrombus was performed by means of lower limb duplex ultrasonography, during which a 46.9×44.7 mm right popliteal artery aneurysm (PAA) was identified (Figure 1). A computed tomography image is presented in Figure 2. He did not have a contralateral PAA. He did not complain of symptoms, such as intermittent claudication, compressive sensations, or pain, and was therefore diagnosed with an asymptomatic PAA. We decided to perform open repair of the right PAA before symptoms developed.

**Figure 3:**
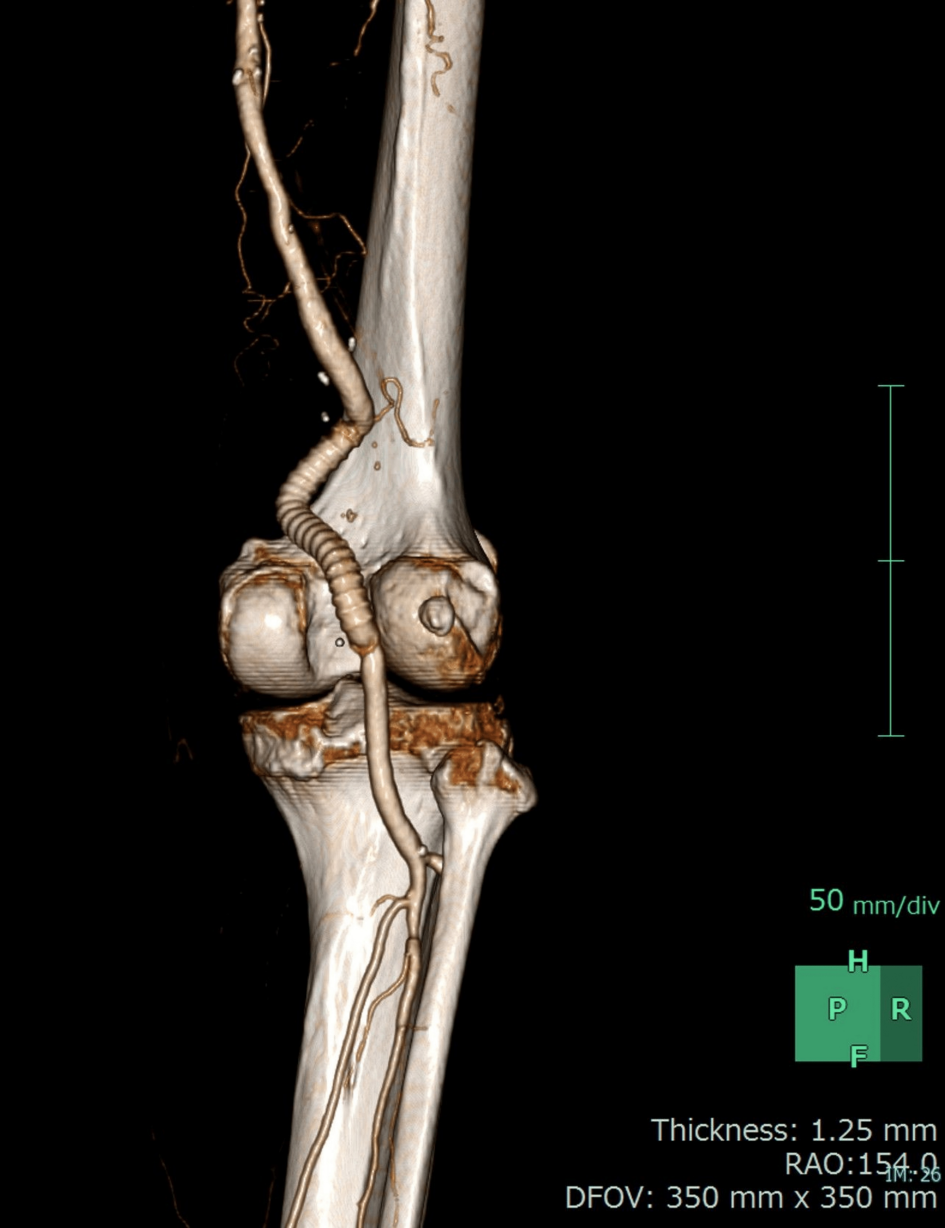
The postoperative three-dimensional computed tomography image showing the reconstruction using a prosthetic graft.

The patient was placed in a prone position, and a posterior approach was used. The aneurysm was opened, and a large amount of thrombus and plaque was observed. We placed an 8 mm Gore Propaten (Flagstaff, AZ: W.L. Gore & Associates, Inc.) interposition graft (Figure 3). Embolism did not occur during the procedure or the perioperative period. Screening for other arterial aneurysms by computed tomography revealed a 47.0 mm abdominal aortic aneurysm. Therefore, after the surgical treatment of the right PAA, the patient underwent endovascular aortic repair. His postoperative course was uneventful following both procedures. The patient remained free of symptoms and complications. At 12-month follow-up, ultrasound assessment confirmed that the graft remained patent and free of anastomotic stenosis.

At our institution, 2,264 patients underwent screening for deep venous thrombus because of abnormal D-dimer concentrations identified prior to surgical procedures under general anesthesia between 2019 and 2023, including in other departments. The present patient was the only one in whom asymptomatic PAA was identified ultrasonographically during screening for deep venous thrombus.

## Discussion

PAA is often diagnosed following the identification of acute lower limb ischemia, and the prevalence of major amputation has been reported to be approximately 20% [3,4]. The Society for Vascular Surgery guidelines recommend that patients with an asymptomatic PAA ≥20 mm in diameter should undergo repair [1]. However, a study reported that even small PAAs were associated with a considerable incidence of thrombosis, clinical symptoms, and distal occlusive disease [6]. The outcomes associated with the surgical management of asymptomatic PAA have been shown to be superior to those for symptomatic aneurysm [2]. Therefore, it is considered that in cases with intraluminal thrombus, asymptomatic PAAs <20 mm may warrant earlier repair [6]. It is essential to recognize PAA, even in the absence of symptoms and regardless of its size.

The prevalence of PAA is estimated to be 0.1-1% in the general population, making it a relatively rare disease [1]. In a review of multiple studies, approximately 40% of patients were asymptomatic at the time of repair [7]. The present case illustrates the rarity of asymptomatic PAA and the difficulties associated with its diagnosis, as previously reported. The gold-standard method of screening for PAA is considered to be duplex ultrasonography for men with large abdominal aortic aneurysms [1]. In addition, we have adopted a protocol in which we screen for deep venous thrombosis when patients have abnormal circulating D-dimer concentrations on preoperative blood testing at our institution (reference range: <0.5 µg/mL). If a PAA of ≥20 mm in diameter is identified incidentally on duplex ultrasonography, it is reported, and the referring physician is advised to consult with a vascular surgeon.

The long-term durability of open surgical repair for PAA has been well-demonstrated in previous studies [2,3,8]. It is reported that endovascular treatment is less invasive and potentially less morbid [9]. Nevertheless, it remains uncertain which modality provides superior long-term patency [9,10]. In the present case, open repair was selected because the patient was expected to have a relatively long life expectancy.

## Conclusions

Here, we have reported the incidental identification of an asymptomatic PAA during ultrasonographic screening for deep venous thrombus. This case underscores the importance of considering PAA detection even in examinations primarily intended for other vascular diseases. Moreover, it highlights the clinical significance of early surgical intervention in asymptomatic PAA to prevent thrombotic or ischemic complications.
